# Xpert®MTB/RIF for the Diagnosis of Tuberculosis in a Remote Arctic Setting: Impact on Cost and Time to Treatment Initiation

**DOI:** 10.1371/journal.pone.0150119

**Published:** 2016-03-18

**Authors:** Olivia Oxlade, Jordan Sugarman, Gonzalo G. Alvarez, Madhukar Pai, Kevin Schwartzman

**Affiliations:** 1 Respiratory Epidemiology and Clinical Research Unit, Department of Epidemiology, McGill University, Montreal, QC, Canada; 2 McGill International Tuberculosis Centre, McGill University, Montreal, QC, Canada; 3 Clinical Epidemiology, The Ottawa Hospital Research Institute, Ottawa, ON, Canada; 4 Division of Respirology, Department of Medicine, University of Ottawa, Ottawa, ON, Canada; London School of Hygiene and Tropical Medicine, UNITED KINGDOM

## Abstract

**Background:**

Tuberculosis (TB) remains a significant health problem in the Canadian Arctic. Substantial health system delays in TB diagnosis can occur, in part due to the lack of capacity for onsite microbiologic testing. A study recently evaluated the yield and impact of a rapid automated PCR test (Xpert®MTB/RIF) for the diagnosis of TB in Iqaluit (Nunavut). We conducted an economic analysis to evaluate the expected cost relative to the expected reduction in time to treatment initiation, with the addition of Xpert®MTB/RIF to the current diagnostic and treatment algorithms used in this setting.

**Methods:**

A decision analysis model compared current microbiologic testing to a scenario where Xpert®MTB/RIF was added to the current diagnostic algorithm for active TB, and incorporated costs and clinical endpoints from the Iqaluit study. Several sensitivity analyses that considered alternative use were also considered. We estimated days to TB diagnosis and treatment initiation, health system costs, and the incremental cost per treatment day gained for each individual evaluated for possible TB.

**Results:**

With the addition of Xpert®MTB/RIF, costs increased while days to TB treatment initiation were reduced. The incremental cost per treatment day gained (per individual investigated for TB) was $164 (95% uncertainty range $85, $452). In a sensitivity analysis that considered hospital discharge after a single negative Xpert®MTB/RIF, the Xpert®MTB/RIF scenario was cost saving.

**Interpretation:**

Adding Xpert®MTB/RIF to the current diagnostic algorithm for TB in Nunavut appears to reduce time to diagnosis and treatment at reasonable cost. It may be especially well suited to overcome some of the other logistical barriers that are unique to this and other remote communities.

## Introduction

Tuberculosis (TB) remains a significant health problem in the Canadian Arctic; in 2012, incidence rates for all forms of TB in Nunavut were 234/100,000, nearly 60 times higher than rates in southern Canada [1]. The reasons for this high TB burden are multi-faceted, reflecting many overlapping risk factors. One factor is health system delays in diagnosis that can occur due to limited capacity for on-site microbiologic testing at the hospital in Iqaluit, the only hospital in the Qikiqtaaluk (Baffin) region of Nunavut. All microbiologic specimens for investigation of possible TB from Qikiqtaaluk (Baffin) are prepared at the hospital and flown South to Ottawa, Ontario, for smears, cultures, and drug susceptibility testing. This contributes to delays in diagnosis and treatment initiation, as well as high costs.

In 2012, the World Health Organization issued an updated strong recommendation for using Xpert®MTB/RIF (Cepheid Inc, Sunnyvale, CA), a fully automated TB diagnostic molecular test, as the initial test for diagnosis of pulmonary TB in certain populations [2]. The Xpert®MTB/RIF (approved by Health Canada in 2012 and the United States Food and Drug Administration in 2013), has high specificity and sensitivity [3], a rapid turnaround time (which ideally translates to faster time to treatment initiation), and minimal bio-safety requirements and training needs. Several studies have considered programmatic and patient benefits from management decisions that reflected Xpert®MTB/RIF test results. One suggested that use of Xpert®MTB/RIF reduced potentially unnecessary empiric therapy [4]; another reported that one negative Xpert®MTB/RIF result can reduce unnecessary and expensive hospital isolation for inpatients initially labelled as possibly having active TB [5].

Based on the potential merits of Xpert®MTB/RIF, a study to evaluate its yield and impact in Nunavut was recently completed [6]. The study showed test sensitivity and specificity were high at 85% and 99% respectively, and time to treatment initiation was significantly shortened, particularly for smear-negative, culture-positive cases, which comprise 2/3 of cases in Nunavut [6]. Given these findings, there are suggestions to incorporate the Xpert®MTB/RIF test into routine practice in Nunavut, consistent with the Canadian TB Standards [6,7]. Xpert®MTB/RIF implementation will require substantial resources for equipment, supplies, and laboratory technician time. In view of the unique epidemiologic, sociodemographic and geographic features of Nunavut, we conducted an economic analysis to evaluate expected costs and changes in times to diagnosis and treatment initiation attributable to adding Xpert®MTB/RIF to diagnostic and TB management algorithms in Nunavut. We also considered costs and the impact on time to treatment initiation of potential changes to TB program policies arising from Xpert®MTB/RIF implementation.

## Methods

### General Description of Model

A deterministic decision analysis model was developed using Tree Age software (Tree Age Pro 2012, Williamstown, MA, USA). We considered a hypothetical cohort of individuals evaluated for possible TB within the Qikiqtaaluk (Baffin) region. We simulated expected events and costs, with and without the integration of Xpert®MTB/RIF into the routine testing algorithm for diagnosis and treatment of active TB. Simulation began with initial contact at a local clinic, and ended at completion of treatment for active TB, or at the end of investigation for those found not to have active TB. Model outputs for each scenario were: 1) days from first presentation to health services to the start of TB treatment and 2) health system costs for all investigations and treatment for suspected or confirmed active TB. Use of Xpert®MTB/RIF to detect rifampin-resistant TB (marker of multi-drug resistance) was not considered, since all TB isolates tested in Nunavut were susceptible to all first line drugs in 2013, and no MDR TB cases were identified in Nunavut from 2003 to 2013 [8].

The mean interval (in days) from initial evaluation to treatment initiation were calculated for: 1) the full cohort of individuals evaluated for TB (calculation includes contribution from both confirmed TB cases, and those who do not have TB and therefore contribute 0 days to the time between initial evaluation and treatment); and 2) for microbiologically confirmed cases only. The incremental cost per treatment day gained (per individual evaluated for TB), was used to summarize the cost relative to the clinical impact of the strategy with Xpert®MTB/RIF added, versus the status quo where only smear and culture are used. The analysis was conducted from the perspective of the health system. No discounting was used, due to the short timeframe.

### Study Population

A hypothetical cohort of persons evaluated for active TB was considered; the prevalence of TB in this group was determined from the study of Xpert®MTB/RIF in Nunavut [6]. Two types of settings were considered in order to capture differences in cost and clinical management of persons evaluated for TB in Qikiqtaaluk (Baffin): 1) Iqaluit (i.e., territorial capital with a hospital) and 2) remote communities (nursing station- no hospital).

### Diagnostic Strategies

**Fig 1:**
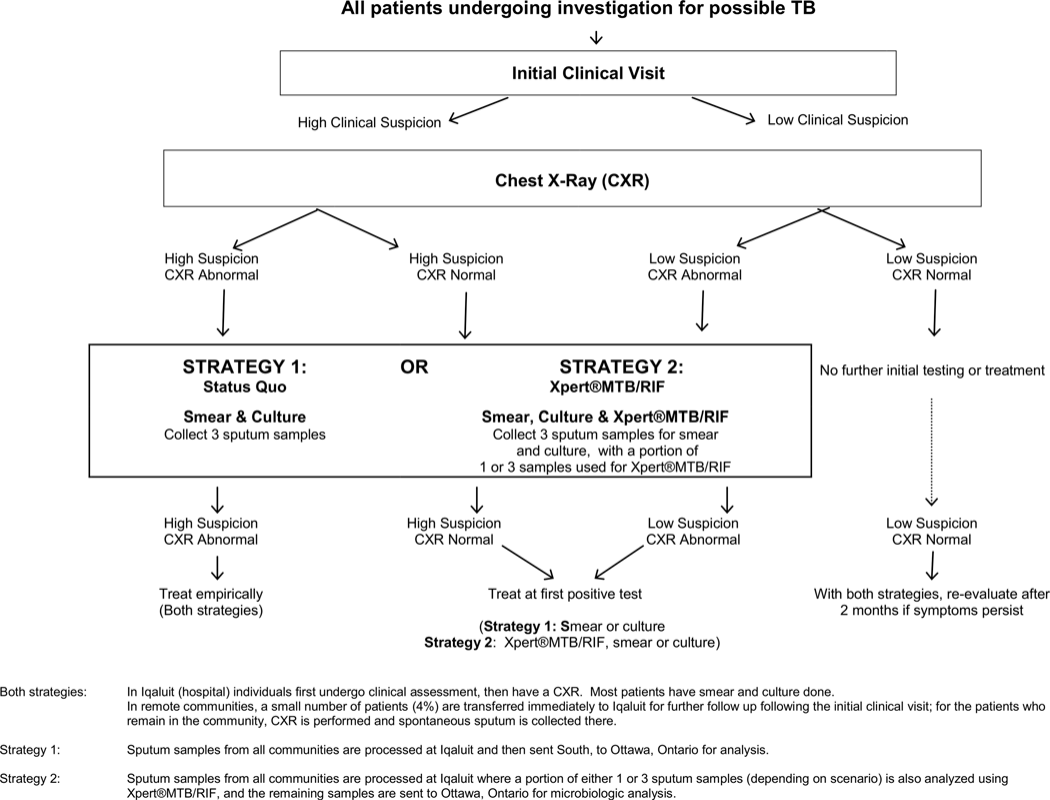
Testing algorithms with the two strategies: Status Quo and Xpert®MTB/RIF.

Two diagnostic strategies were considered. The testing algorithms for each strategy are illustrated in Fig 1. With **Strategy 1**, routine tests for TB diagnosis included smear and culture, both performed in Ottawa. With **Strategy 2**, Xpert®MTB/RIF performed in Iqaluit was added to the testing algorithm. With both strategies, persons in Iqaluit with a high clinical suspicion for active TB and a suggestive chest X-ray, were hospitalized for empiric treatment initiation and microbiologic testing. Similarly, with both strategies, persons living in remote communities with a high clinical suspicion for active TB, and a suggestive chest X-ray, began empiric treatment in their communities. Hence for such individuals, Xpert®MTB/RIF results could not advance treatment initiation. However, positive Xpert®MTB/RIF results led to earlier treatment for others whose clinical and radiographic findings did not warrant immediate empiric treatment. Details about these diagnostic strategies and the yield of the various tests are provided in an on-line appendix (See S1 File). Relevant clinical and epidemiolgoic parameters, as well as intervals (in days) between milestones in the diagnostic algorithms are listed in Tables 1 and 2.

**Table 1:** Clinical and Epidemiologic Probabilities Associated with TB Investigation and Care in Nunavut, Canada.

Description	Value	Range	Source
Proportion of Nunavut population that lives in Iqaluit	21.0%	—	[17]
Prevalence of active TB among those who present to clinic for TB evaluation	27/344 = 7.8%	5.0–10.6	[6]
Smear result among persons with active TB			[7]
smear positive	32.1%	23.5–40.8	
smear negative	67.9%	-	
Sensitivity of relying on clinical symptoms for initial TB evaluation	61.0%	0.51–0.71	[18]
Specificity of relying on clinical symptoms for initial TB evaluation	83.0%	0.82–0.84	[18]
Sensitivity of chest x-ray	90.5%	90.0–100	[1, 19]
Specificity of chest x-ray	60.0%	60.0–70.0	[1, 19]
Probability of producing suitable sputum samples given CXR abnormality	81.2%	74.7–89.6	[20]
Probability of producing suitable sputum samples given no CXR abnormality	50%	0–100	[21]
Probability of an individual being evaluated for TB in a remote community being sent to Iqaluit for further TB related clinical evaluation	4.0%	0–5.0	Personal communication Van Dyk
Anti-tuberculosis treatment outcomes			[22]
Cure	94.6%	94.2–96.8	
Failure	1.2%		
Death	4.2%	2.3–6.2	
Sensitivity of Xpert®MTB/RIF for smear positive TB	95.0%	85.0–1.00	[6]
Sensitivity of Xpert®MTB/RIF for smear negative TB	57.0%	20.4–93.6	[6]
Specificity of Xpert®MTB/RIF	99.0%	98.0–1.00	[6]

**Table 2:** Intervals for Patients Ultimately Diagnosed with TB disease, From Entry to Health System to Eventual TB Diagnosis and Treatment Initiation in Nunavut, Canada.

Interval prior to diagnosis	Average Days	Range	Reference
**IQALUIT**			
Interval between ordering CXR and result provided to doctor	1.5	1.1, 1.9	Personal communication Van Dyk
Interval between obtaining 3 spontaneous sputum samples and lab stamp in Iqaluit	7	5.3, 8.8	Personal communication, Alvarez and DeMaio
Interval between obtaining 3 sputum samples via induction in Iqaluit and lab stamp in Iqaluit	3	2.3, 3.8	Personal communication, Alvarez and DeMaio
Interval between sending out all 3 sputum samples from Iqaluit to smear result and first dose of meds when required (regardless of setting)	7.7	5.8, 9.6	[6]
Interval between sending out 3 sputum samples from Iqaluit to culture result and first dose of meds when required (regardless of setting)	37.1	27.8, 46.4	[6]
**REMOTE COMMUNITY**			
Interval between ordering CXR and result provided to doctor	4	3.0, 5.0	Personal communication Van Dyk
Interval between obtaining 3 spontaneous sputum samples and lab stamp in Iqaluit (2 extra days added for shipment of sample to Iqaluit)	9	6.8, 11.3	Personal communication, Alvarez and DeMaio
Interval in remote communities if spontaneous sputum cannot initially be obtained (2nd attempt after 2 weeks is assumed to be successful)	14	10.5, 17.5	Assumption
**XPERT®MTB/RIF**			
Interval between obtaining single sputum sample (either spontaneous or induced) for Xpert®MTB/RIF and lab stamp in Iqaluit	1	0.8, 1.3	Assumes one day for collection of single sample to use for Xpert®MTB/RIF
Interval between obtaining single sputum sample for Xpert®MTB/RIF and lab stamp in remote community	3	2.3, 3.8	Assumes one day for collection of single sample to use for Xpert®MTB/RIF, plus 2 days travel
Interval between lab stamp of receipt of single sputum sample to Xpert result and first treatment doses (if needed) in I Iqaluit	1.8	1.4, 2.3	[6]
Interval between lab stamp of receipt of single sputum sample to Xpert result and first treatment dose (if needed) in remote community (2 extra days added for shipment of sample to Iqaluit)	3.8	2.9, 4.8	Assumption
**MISSED CASE**			
Interval associated with missing a case	60	45.0, 75.0	Assumption

FOOTNOTE: Table shows the average number of days taken to complete each activity, according to information obtained from Nunavut sources, or assumption when no estimate available.

### Costing

TB-related health system costs included materials, shipping, patient transport, fourteen days hospitalization at Qikiqtani General Hospital (based on information provided by the Nunavut Health Department) and professional wages. Nunavut physician fees were obtained from the Government of Nunavut Physician Services Department; Ontario physician fees were obtained from the 2013 Ontario Health Insurance Plan Fee Schedule. Component costs and total costs for the status quo strategy (Strategy 1) are listed in Table 3. Additional costs for the Xpert®MTB/RIF strategy are listed in Tables 4 and 5. Machine costs (calibration, cartridges, shipping) and salary for the laboratory technologist working with specimens were included. Costs associated with unreadable or faulty Xpert®MTB/RIF modules were not included, as none were reported in the Iqaluit pilot study. In the base case, the cost per specimen tested using Xpert®MTB/RIF was $133.03, assuming 1651 specimens analyzed per year (the number reported for Nunavut in 2013 by the Nunavut Health Department). All costs were reported in 2014 Canadian dollars. Costs from 2013 were inflated using inflation rates from the Bank of Canada [9].

**Table 3:** Costs Associated with TB investigation and Care in Nunavut, Canada. (2014 $CAD).

Description	Value (2014 $CAD)	Source
Initial TB assessment Iqaluit (1 MD visit)	**64.49**	Nunavut Health Department
Chest x-ray in Nunavut in a hospital or remote community	**67.32**	
-Technician Fee: 15-min appointment	10.14	Qikiqtani General Hospital
-Two films (two views)	6.13	Qikiqtani General Hospital
-Radiologic Interpretation in Ottawa	51.06	[23]
Spontaneous sputum collection Nunavut in a hospital or remote community	**3.49**	
-Sending South	3.02	First Air Cargo Division
-Cups	0.47	Fischer Scientific
Sputum induction in Iqaluit	**96.00**	[21]
Analysis of sputum		
If result is negative	**29.04**	
-Base sputum analysis fee for 3 samples	29.04	Gamma Dynacare Ottawa
If result is positive	**80.09**	
-Base sputum analysis fee for 3 samples	29.04	Gamma Dynacare Ottawa
-1 PCR probe if positive	51.05	Gamma Dynacare Ottawa
Hospitalization per day in Iqaluit, Nunavut	**2463.23**	
-Hospitalization Qikiqtani General Hospital	2463.23	Nunavut Health Department
Mean Days spent in hospital for TB treatment initiation in Iqaluit, Nunavut	14	Nunavut Health Department
Initial TB assessment in a remote community in Nunavut	**23.83**	
-Nurse fee for TB assessment (20 mins / assessment at 70.01/hr—range for hourly wage 52.74–87.30)	23.83	[24]
Administering DOT in Nunavut	**1410.04**	
-Nurse fee for 104 DOT administrations (10 mins/visit)	1410.04	[24]
Drugs for TB therapy	**432.15**	Qikiqtani General Hospital
Follow up following treatment initiation	**146.27**	
-5 visits with Nurse (20 mins/visit)	119.16	[24]
-5 Liver Function Tests (5.31 each)	27.11	Nunavut Health Department
From remote community to Iqaluit, Medical Evacuation	**10000**	Assumption
From Iqaluit to remote community, flight home	**1428.83**	First Air (Average common return fare to/from Iqaluit)

**Table 4:** Costs Associated with Xpert®MTB/RIF.

Cost Component	Base Case Cost (2014 $CAD)	Range	Reference
Gene Xpert®MTB/RIF 4 Module, machine with laptop and printer	$60,000	$30,000-$90,000	Inter Medico QC*
Life expectancy of machine	10 years	-	Inter Medico QC
Depreciated annual machine cost assuming 10 year life expectancy	$6000	-	Calculated
Gene Xpert®MTB/RIF Cartridges	$60/cartridge + $2.50 shipping	$35-$60	Inter Medico QC
Calibration Module	$450/year	-	Inter Medico QC
Laboratory Technologist dedicated to Xpert®MTB/RIF	$110,000 annual salary (includes benefits)	-	[25, 26]

* Inter Medico is the official distributor for Cepheid/ Xpert®MTB/RIF in Canada

**Table 5:** Per Sample Component Costs for Xpert®MTB/RIF, by Number of Individuals and Samples Evaluated.

Cost Component (per sample)	Cost for 1651 Individuals 1 sample per individual*	Cost for 1651 Individuals 3 samples per individual**
Machine Cost (Calculated using base case depreciated machine cost shown above)	$3.63	$1.21
Gene Xpert®MTB/RIF Cartridges	$62.50	$62.50
Calibration Module	$0.27	$0.09
Laboratory Technologist	$66.63	$22.21
**Total per Sample**	**$133.03**	**$86.01**
**Total per Individual Investigated**	**$133.03**	**$258.03**

* Cost used in base case analysis as well as Sensitivity Analysis 2a (Hospital discharge with 1 negative test)

** Cost used in Sensitivity Analysis 1 (3 sputum samples per individual) and Sensitivity Analysis 2b (Hospital discharge with 3 negative tests)

### Sensitivity Analysis

Two additional scenarios for use of Xpert®MTB/RIF were considered as part of our sensitivity analysis:

#### 1) Three Sputum Samples per Individual

Extracts from 3 sputum samples were analyzed using Xpert®MTB/RIF (vs 1 in the base case). Cost per sample analyzed was reduced, but test cost per individual was increased (Table 5). Based on 3 samples, test sensitivity increased to 99.8% for smear-positive cases and 90.2% for smear-negatives [10].

#### 2) Hospital Discharge with Negative Tests

Individuals hospitalized for empiric treatment initiation without microbiologic confirmation, were discharged from hospital 24 hours after receipt of a negative Xpert®MTB/RIF result from either, a) one Xpert®MTB/RIF test or, b) 3 consecutive Xpert®MTB/RIF tests. However, outpatient treatment and associated costs continued until a negative culture results were obtained (ie. after two months).

### Univariate Sensitivity Analysis

Univariate sensitivity analysis was conducted to understand the influence of key variables on projected costs and days to treatment initiation. Given uncertainty in point estimates, the following were varied extensively: 1) Costs per sample analyzed with Xpert®MTB/RIF (total cost including cartridge, machine, labor from $50-$350); 2) Proportion of individuals under investigation in remote communities moved to Iqaluit hospital for further TB work up (from 0%-10%); 3) Underlying prevalence of TB disease in those evaluated for possible TB (from 0%-20%), as this likely varies within the region; and 4) Cost for medical evacuation (from $8,000-$12,000).

### Probabilistic Sensitivity Analysis

Finally, probabilistic sensitivity analysis was conducted using 1,000 Monte Carlo trials, to obtain a 95% uncertainty range (2.5^th^ and 97.5^th^ percentiles) around the point estimates for projected outcomes for each strategy. Distributions were defined for probabilities, and times to diagnosis and treatment milestones. Costs were not varied as most values were provided from reliable sources. For most probabilities used in the model, beta distributions were fitted to 95% confidence intervals obtained from empiric data. For times to diagnosis and treatment inputs, a range of +/- 25% of the point estimate was used, as empiric data were not always available. Triangular distributions were fitted using these maximum and minimum estimates. More detail on distributions is provided in appendix (Tables A and B in S1 File).

## Results

In the base case scenario, with the status quo strategy, total cost per individual evaluated for TB for the full population (Iqaluit and remote communities combined) was $2278 (95% uncertainty range (UR): $1668, $2649). The mean days to treatment initiation was 1.7 (0.7, 3.0) per person evaluated for possible TB, and 21.9 (7.9, 36.8) per confirmed active TB case (Table 6). Costs were highest in the Iqaluit hospital community $5261 ($3397, $6815) mainly due to hospitalization, however, time to treatment initiation was shorter because the testing process was faster.

**Table 6:** Base Case Analysis: Projected Cost and Time to Treatment Initiation, Incremental Outcomes and Incremental Cost per Treatment Day Gained in Nunavut, Canada.

	Status Quo			Gene Xpert®MTB/RIF Added			Gene Xpert®MTB/RIF vs Status Quo			
	$ per Individual evaluated for TB	Time to treatment initiation per individual evaluated for TB	Time to treatment initiation per confirmed TB case	$ per Individual Evaluated for TB	Time to treatment initiation per individual evaluated for TB	Time to treatment initiation per confirmed TB case	Incremental cst	Treatment day gained per individual evaluated for TB	Treatment day gained per confirmed TB case	Incremental cost per treatment day gained (per individual evaluated for TB)
Iqaluit Hospital Community	5261	1.3	16.5	5455	0.7	9.2	194	0.6	7.29	340
Remote Nunavut Community	1486	1.8	23.4	157	1.2	15.4	90	0.6	7.99	145
**Full Cohort**	2278	1.7	21.9	2390	1.1	14.1	100	0.6	7.83	164

With the addition of Xpert®MTB/RIF, overall costs increased by roughly $100 per individual investigated, while days to treatment initiation were reduced by 0.6 days per person evaluated for possible TB, and by 7.8 days per confirmed TB case. The estimated incremental cost per treatment day gained (per individual evaluated for TB) was $164 ($85, $452) (Table 6). In the Iqaluit hospital community, the estimated incremental cost per treatment day gained (per individual evaluated for TB) was higher at $340 ($137, $1141), while in the remote community it was $145 ($72, $392).

### Sensitivity Analyses

When 3 sputum specimens were analyzed with Xpert®MTB/RIF (vs 1 in the baseline scenario) (Table 7), costs were higher, but time to treatment initiation was further reduced because of increased sensitivity with 3 samples. The estimated incremental cost per treatment day gained (per individual evaluated for TB) was $197, a small change compared to $164 for a single sample.

**Table 7:** Sensitivity Analysis: Projected Cost and Time to Treatment Initiation, Incremental Cost (savings) and Treatment Days Gained, and Incremental Cost per Treatment Days Gained, per Individual Evaluated for TB, in Nunavut, Canada.

	Status Quo		Gene Xpert®MTB/RIF Added		Gene Xpert®MTB/RIF vs Status Quo		
	$ per Individual evaluated for TB	Time to treatment initiation	$ per Individual evaluated for TB	Time to treatment initiation	*Incremental cost	Treatment days gained	Incremental cost per treatment day gained
**Base Case**							
Iqaluit Hospital Community	5261	1.3	5455	0.7	194	0.6	340
Remote Nunavut Community	1486	1.8	1576	1.2	90	0.6	145
**Full Cohort**	2278	1.7	2390	1.1	100	0.6	164
**Sensitivity Analysis 1: 3 specimens analyzed with Xpert®MTB/RIF per individual**							
Iqaluit Hospital Community	5261	1.3	5510	0.5	249	0.8	304
Remote Nunavut Community	1486	1.8	1633	1.0	147	0.9	169
**Full Cohort**	2278	1.7	2447	0.9	169	0.9	197
**Sensitivity Analysis 2a: Hospital discharge for those started on empiric therapy who are Xpert®MTB/RIF negative- 1 specimen analyzed with Xpert®MTB/RIF**							
Iqaluit Hospital Community	5261	1.3	2960	0.7	-2301	0.6	Saving
Remote Nunavut Community	1486	1.8	982	1.2	-504	0.6	Saving
**Full Cohort**	2278	1.7	1397	1.1	-881	0.6	Saving
**Sensitivity Analysis 2b: Hospital discharge for those started on empiric therapy who are Xpert®MTB/RIF negative- 3 specimens analyzed with Xpert®MTB/RIF**							
Iqaluit Hospital Community	5261	1.3	3349	0.5	-1912	0.8	Saving
Remote Nunavut Community	1486	1.8	1061	1.0	-425	0.9	Saving
**Full Cohort**	2278	1.7)	1541	0.9	-727	0.9	Saving

* Negative incremental cost indicates "Savings" with Xpert®MTB/RIF strategy relative to the Status Quo strategy

In the scenario with hospital discharge after one negative Xpert result (Table 7), costs for the Xpert®MTB/RIF scenario were substantially reduced, and it became cost saving in all settings. For the entire cohort, costs per individual investigated were reduced by almost $1000. In Iqaluit, costs were reduced by $2495 per person, because all individuals initiated treatment in hospital. In the remote communities, hospital release following one negative Xpert®MTB/RIF had much less impact as only 4% of individuals under investigation were transferred to the Iqaluit hospital. Days to treatment initiation remained the same as in the base case scenario, as they were unrelated to hospital discharge.

If three negative Xpert®MTB/RIF results were required for hospital release (Table 7), savings were still projected relative to the status quo.

### Univariate Sensitivity Analysis

**Fig 2:**
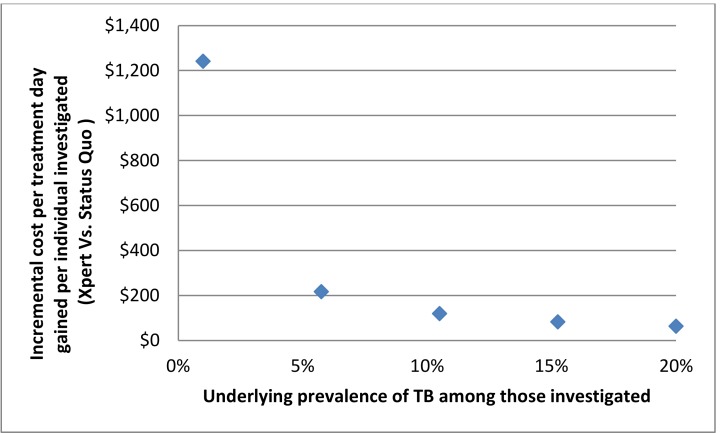
Underlying Prevalence of TB vs. Incremental Cost per Treatment Day Gained.

When the total "all in" costs per sample analyzed using Xpert®MTB/RIF were varied from $50 to as high as $350, costs per day of earlier treatment initiation per individual investigated for TB increased proportionately. With a high-end cost of $350 per sample analyzed, the incremental cost per day gained was approximately $400. When the proportion of individuals transported to Iqaluit hospital for further investigation was varied from 0–10%, the effect on the incremental cost per treatment day gained for the Xpert®MTB/RIF vs status quo was minimal, increasing from $144 to $187. The effect of varying the underlying prevalence of TB among those referred for investigation was more substantial. Increasing the prevalence from 1% to 20% decreased the incremental cost per treatment day gained from over $1000 to approximately $60 (Fig 2). Increasing the cost for medical evacuation from $8,000-$12,000 increased projected costs only by a small amount because of the small number of people who required evacuation.

Additional results from the probabilistic sensitivity analysis are shown in Table C in S1 File.

### Interpretation

Adding Xpert®MTB/RIF to the current Nunavut diagnostic algorithm for TB is likely to be cost effective. The estimated reduction in time to treatment initiation was substantial, 7.8 days per confirmed active case, and the incremental cost per treatment day gained was only $164, versus the status quo scenario where only smear and culture are used for microbiologic diagnosis of active TB.

Xpert®MTB/RIF has been judged cost effective in other settings, both high income [11] and low income [12–14]. The health care infrastructure and clinical practices in place prior to Xpert®MTB/RIF implementation were important determinants of study findings in such settings. The clinical care landscape in the Canadian Arctic is unique. It is a remote environment with enormous distances between communities, where healthcare and laboratory infrastructure is sparse, and clinical samples must be shipped to southern Canada for microbiological confirmation. Consequently, TB patients are often only diagnosed and treated after some delay, and may therefore have more advanced disease, in contrast to high income urban settings where Xpert®MTB/RIF use has been studied, such as San Francisco [4] and Montreal [15]. In Montreal, where Xpert®MTB/RIF appeared to have particularly limited utility, most individuals investigated for TB were asymptomatic and referred via immigration screening [15], which differs substantially from the Arctic setting. Hence, because of the differences in infrastructure and patient characteristics, Xpert®MTB/RIF may be particularly valuable in the Canadian Arctic. The Canadian TB Standards recommend that where there is no on-site capacity for smear microscopy and culture, an automated test can be used to make rapid decisions about TB treatment and isolation, pending routine smear and culture results [7].

Our analysis focused on the Qikiqtaaluk (Baffin) region, where the great majority of Nunavut TB cases are found. We had to make some generalizations about clinical decision making in our study. Wherever possible, the likelihood of clinical events was informed by Nunavut policy recommendations, or by input from clinicians with extensive experience in the investigation of possible TB in Nunavut. Study inputs were relevant and accurate; most, including test performance, epidemiologic and costing data came from the study of Xpert®MTB/RIF in Nunavut [6]. We did not account for secondary transmission from infectious TB patients. The greatest reduction in diagnostic delays involved smear-negative patients, for whom the probability of onward transmission is substantially lower than with smear-positive disease [16]; their effect would likely be relatively small at a population level [13].

Costs were considered only from a health system perspective, we did not address those borne by patients and families. Costs were limited to TB diagnosis and treatment; downstream costs such as contact investigation were not considered. There was some uncertainty as to the number of sputum samples to be analyzed using Xpert® RIF/MTB, therefore we considered scenarios where either 1 or 3 samples per individual were analyzed. There was also uncertainty about the placement of the Xpert machine; placing the machine in more remote communities could further reduce delays and costs by eliminating the transfer of specimens to Iqaluit. We did not evaluate the logistics and costs of on-site Xpert®MTB/RIF testing in remote communities.

A major strength of our study is the integration of clinical and cost data from a field evaluation of Xpert®MTB/RIF in Iqaluit. Wherever possible, Nunavut data and experience informed our modelling. We focused on current practice and examined potential practice changes as a consequence of Xpert®MTB/RIF adoption.

In conclusion, Xpert®MTB/RIF appears potentially cost effective as an addition to the diagnostic algorithm in Nunavut. Although we focused on the Qikiqtaaluk (Baffin) region, our study provides an in-depth analysis of the cost and potential yield of Xpert®MTB/RIF in the Canadian Arctic, and considers possible scenarios for its use in guiding clinical management. The assessment of cost and clinical impact, and of scenarios for optimal use, may be relevant for other remote communities where there is ongoing TB-related morbidity, and where it is proposed for on-site TB diagnosis. Xpert®MTB/RIF may be especially well suited to overcome some of the logistical barriers unique to this remote Arctic setting.

## Supporting Information

S1 File Supplemental Methods -Details Of Diagnostic Strategies & Supplemental Results. **Table A.** Range and Distribution Around Point Estimate of Clinical and Epidemiologic Model Probabilities. **Table B.** Range and Distribution Around Point Estimate of Average Intervals to Diagnosis Associated with Tuberculosis Care. **Table C.** Base Case and Sensitivity Analysis with 95% Uncertainty ranges: Projected Cost and Time to treatment initiation, Incremental Cost (savings) and treatment days gained, and Cost per Treatment days gained, per Individual Evaluated for TB in Nunavut, Canada.
(DOCX)
