# Supplementary material for: Xpert®MTB/RIF for the Diagnosis of Tuberculosis in a Remote Arctic Setting: Impact on Cost and Time to Treatment Initiation
Source: PLoS One. 2016 Mar 18;11(3):e0150119. doi: 10.1371/journal.pone.0150119 (PMC4798714; doi:10.1371/journal.pone.0150119)
Supplement: S1 File — Table A. Range and Distribution Around Point Estimate of Clinical and Epidemiologic Model Probabilities. Table B. Range and Distribution Around Point Estimate of Average Intervals to Diagnosis Associated with Tuberculosis Care. Table C. Base Case and Sensitivity Analysis with 95% Uncertainty ranges: Projected Cost and Time to treatment initiation, Incremental Cost (savings) and treatment days gained, and Cost per Treatment days gained, per Individual Evaluated for TB in Nunavut, Canada. (DOCX) [file pone.0150119.s001.docx]

**Xpert®MTB/RIF for the diagnosis of tuberculosis in a remote Arctic setting: Impact on cost and time to treatment initiation**

**Supplemental Methods**

**Details Of Diagnostic Strategies**

**Strategy 1: Status Quo TB Diagnostic Algorithms**

**- Investigation for TB in Iqaluit, (Hospital Community) Qikiqtaaluk (Baffin) region of Nunavut**

In the hospital community of Iqaluit, cohort members presented to the local public health clinic for investigation of possible active TB. These individuals were divided into two groups; "high clinical suspicion" or "low clinical suspicion" based on TB related symptoms, socio-demographic risk factors (e.g. known recent TB contact), and other clinical indices. In our model, 61% of individuals with underlying TB were classified as high clinical suspicion, based on results of a large published study of a clinical symptom screen in persons with possible TB (ie. presence of cough, fever or high sweats >7 days) [1]; 17% of individuals without TB would also be considered high clinical suspicion based on symptom screen [[1](#_ENREF_9)]. Regardless of clinical suspicion, all persons underwent chest X-ray (CXR). CXR had 95% sensitivity and 60% specificity for active TB [[2](#_ENREF_7)]. Following CXR, individuals were classified as having a CXR suggestive of active TB, or not.

All individuals who are classified as high clinical suspicion, with a CXR also suggestive of active TB, are admitted to hospital (for two weeks) and begin empiric treatment, consistent with local policy. Further work up (sputum collection/induction) is also carried out at this time. All microbiologic samples are sent to Ottawa for diagnosis of TB using smear and culture.

Individuals for whom the clinical suspicion is high, but the CXR is not considered suggestive of active TB, undergo sputum collection for smear and culture on an outpatient basis. If they subsequently have a positive sputum smear or culture, they are admitted to hospital for treatment initiation. The same applies to persons with low clinical suspicion, but a CXR suggestive of possible active TB.

Individuals started on empiric therapy because of high clinical and radiographic suspicion receive a full treatment course, i.e. six months, even if cultures are ultimately found to be negative. This reflects current clinical practice in Nunavut, where between 1999 and 2011, 22% of patients who received full treatment for active TB did not have positive microbiologic results (Dr. M Baikie, Medical Officer of Health, Government of Nunavut, personal communication).

Individuals who truly have underlying active TB but classified at the outset as low clinical suspicion with a CXR not suggestive of active TB (less than 0.5% of cases) were not further investigated; they are assumed to wait an average 2 months before seeking care again. At this timepoint we assumed their disease will have progressed, and they will be accurately diagnosed and then hospitalized to begin treatment.

Individuals who do not have underlying TB will follow the same clinical algorithm, as disease status is not known to the treating health care professional. These individuals classified as low clinical suspicion with a CXR not suggestive of active TB will not return for any further TB related investigation.

Key probabilities and assumptions relating to scenarios are provided in Table A.

**Table A. Range and Distribution Around Point Estimate of Clinical and Epidemiologic Model Probabilities**

| **Description** | **Value** | **Range** | **Distribution** |
| --- | --- | --- | --- |
| Proportion of Nunavut population living in a Iqaluit | 21.0 | -- |  |
| Prevalence of active TB among those who present to clinic for TB evaluation(As described in Xpert study) | 7.8 | 5.0-10.6 | Beta (7.5, 90) |
| Smear result among persons with active TB |  |  | Beta (32, 68) |
| smear positive | 32.1 | 23.5 – 40.8 |  |
| smear negative | 67.9 | - |  |
| Sensitivity of relying on clinical symptoms for initial TB screening | 61.0 | 51.0-71.0 | Beta (61, 39) |
| Specificity of relying on clinical symptoms for initial TB screening | 83.0 | 82.0-84.0 | Beta (830, 170) |
| Sensitivity of chest x-ray | 90.5 | 90.0 - 100 | Uniform (0.9, 1) |
| Specificity of chest x-ray | 60.0 | 60.0 - 70.0 | Uniform (0.6, 0.7) |
| Probability of producing suitable sputum samples given CXR abnormality | 81.2 | 74.7 - 89.6 | Beta (81, 19) |
| Probability of producing suitable sputum samples given no CXR abnormality | 50 | 0 - 100 | Triangular (0, 0.5, 1) |
| Probability of a TB suspect in a remote community being sent to Iqaluit for further TB related work up | 4.0 | 2.0 - 5.0 | Beta (37, 960) |
| Anti-tuberculosis treatment outcomes |  |  |  |
| Cure | 94.6 | 94.2 – 96.8 | Beta (946, 54) |
| Failure | 1.2 |  |  |
| Death | 4.2 | 2.3 – 6.2 | Beta (42, 958) |
| Sensitivity of Xpert RIF/MTB for smear positive TB | 95.0 | 85.0-1.00 | Beta (95, 6) |
| Sensitivity of Xpert RIF/MTB for smear negative TB | 57.0 | 20.4-93.6 | Beta (5.7, 4.3) |
| Specificity of Xpert RIF/MTB | 99.0 | 98.0-1.00 | Beta (990, 10) |

**- Investigation for TB in remote communities, Qikiqtaaluk (Baffin) region of Nunavut**

In remote communities in the Qikiqtaaluk (Baffin) region of Nunavut, 4% of all persons investigated for TB are estimated to be transferred to the hospital in Iqaluit via air transport for full TB work-up, because of clinical complexity including co-morbidities. The remainder are investigated and managed locally. Individuals evaluated for TB are classified as high and low suspicion as in the Iqaluit hospital community. All individuals who are high suspicion with a suggestive CXR begin TB treatment empirically in the community, and provide 3 sputum samples for smear and culture. All samples are sent to the Iqaluit hospital and then on to Ottawa for microbiologic confirmation. Any individual who cannot spontaneously produce sputum will wait for 2 weeks, at which point it is assumed he/she will be able to produce sputum spontaneously. Individuals for whom clinical suspicion is low, and/or who do not have a CXR suspicious for active TB, management proceeds as described for Iqaluit, except that days before smear and culture results are available are greater, and treatment is given in the community.

**Strategy 2: Xpert®MTB/RIF TB Diagnostic Algorithms**

The same diagnostic algorithms as in Strategy 1 with the addition of Xpert®MTB/RIF microbiologic test, are used for both Iqaluit and remote communities. Regardless of setting, a portion of one sputum sample per person investigated, is analyzed using Xpert®MTB/RIF in the Iqaluit hospital lab. The remainder of the sputum samples are sent for analysis in Ottawa. The sensitivity and specificity of Xpert®MTB/RIF for TB disease was taken from the Xpert®MTB/RIF study in Nunavut [[3](#_ENREF_6)]; smear-positive disease (94%) and smear-negative disease (57%) will be detected with the analysis of one sputum sample using Xpert®MTB/RIF. Specificity of Xpert®MTB/RIF is 99% [[3](#_ENREF_6)], (Table 1). A positive Xpert®MTB/RIF result leads to treatment initiation in hospital for those not already treated empirically. However, consistent with local policy, a negative Xpert®MTB/RIF result does not result in discontinuation of treatment or discharge from hospital for those already on empiric treatment. Other elements of the diagnostic and treatment algorithm are as for Strategy 1.

Further details about distributions around intervals before diagnosis associated with each test in both Iqaluit and remote communities are provided in Tables S2.

**Table B. Range and Distribution Around Point Estimate of Average Interval to Diagnosis Associated with Tuberculosis Care**

| **Interval prior to diagnosis** | **Average Days** | **Range (+/- 25% of point estimate)** | **Distribution** |
| --- | --- | --- | --- |
| **IQALUIT** | | | |
| Interval between ordering CXR and result provided to MD | 1.5 | 1.1, 1.9 | Triangular (1.1, 1.5 ,1.9) |
| Interval between obtaining 3 spontaneous sputum samples and lab stamp in Iqaluit | 7.0 | 5.3, 8.8 | Triangular (5.3, 7.0, 8.8) |
| Interval between obtaining 3 sputum samples via induction in Iqaluit and lab stamp in Iqaluit | 3.0 | 2.3, 3.8 | Triangular (2.3, 3.0, 3.8) |
| Interval between sending out all 3 sputum samples from Iqaluit to smear result and first dose of meds when required (regardless of setting) | 7.7 | 5.8, 9.6 | Triangular (5.8, 7.7, 9.6) |
| Interval between sending out 3 sputum samples from Iqaluit to culture result and first dose of meds when required (regardless of setting) | 37.1 | 27.8, 46.4 | Triangular (27.8, 37.1, 46.4) |
| **REMOTE COMMUNITY** | | |  |
| Interval between ordering CXR and result provided to MD | 4.0 | 3.0, 5.0 | Triangular (3.0, 4.0, 5.0) |
| Interval between obtaining 3 spontaneous sputum samples and lab stamp in Iqaluit (2 extra days added for shipment of sample to Iqaluit) | 9.0 | 6.8, 11.3 | Triangular (6.8, 9.9, 11.3) |
| **XPERT** | | |  |
| Interval between obtaining single sputum sample (either spontaneous or induced) for Xpert and lab stamp in Iqaluit | 1.0 | 0.8, 1.3 | Triangular (0.8, 1.0, 1.3) |
| Interval between obtaining single sputum sample for Xpert and lab stamp in Remote Community | 3.0 | 2.3, 3.8 | Triangular (2.3, 3.0, 3.8) |
| Interval between lab stamp of receipt of single sputum sample to Gene X result and first dose of meds (if needed) in Iqaluit | 1.8 | 1.4, 2.3 | Triangular (1.4, 1.8, 2.3) |
| Interval between lab stamp of receipt of single sputum sample to Gene X result and first dose of meds (if needed) in Remote Community  (2 extra days added for shipment of sample to Iqaluit) | 3.8 | 2.9, 4.8 | Triangular (2.9, 3.8, 4.8) |
| **MISSED CASE** | | |  |
| Interval associated with missing a case | 60.0 | 45.0, 75.0 | Triangular (45.0, 60.0, 75.0) |

**Supplemental Results:**

**Table C. Base Case and Sensitivity Analysis with 95% Uncertainty ranges: Projected Cost and Time to treatment initiation, Incremental Outcomes and Cost per treatment day gained in Nunavut, Canada**

|  | **Status Quo** | | **Gene Xpert®MTB/RIF Added** | | **Gene Xpert®MTB/RIF vs Status Quo** | | |
| --- | --- | --- | --- | --- | --- | --- | --- |
|  | $ per Individual Evaluated for TB | **Time to treatment initiation** per Individual Evaluated for TB | $ per Individual Evaluated for TB | **Time to treatment initiation** per Individual Evaluated for TB | *Incremental Cost | **Treatment days gained** per Individual Evaluated for TB | Cost per Treatment days gained (per Individual Evaluated for TB) |
| **Base Case** | | | | | | | |
| Iqaluit Hospital Community | 5261 (3397, 6815) | 1.28 (0.45, 2.13) | 5455 (3577, 6999) | 0.71 (0.24, 1.21) | 194 (112, 285) | 0.57 (0.18-1.06) | 340 (137, 1141) |
| Remote Nunavut Community | 1486 (1019, 1656) | 1.82 (0.66, 3.12) | 1576 (1099, 1736) | 1.20 (0.42, 2.04) | 90 (74, 97) | 0.62 (0.21-1.21) | 145 (72, 392) |
| **Full Cohort** | 2278 (1668, 2649) | 1.71 (0.67, 3.01) | 2390 (1704, 2762) | 1.10 (0.40, 1.91) | 100 (82, 133) | 0.61 (0.22, 1.19) | 164 (85, 452) |
| **Sensitivity Analysis 1: 3 specimens analyzed with Xpert®MTB/RIF per individual** | | | | | | | |
| Iqaluit Hospital Community | 5261 (3419, 7010) | 1.28 (0.48, 2.26) | 5510 (3624, 7260) | 0.46 (0.14, 0.71) | 249 (163, 324) | 0.82 (0.32, 1.53) | 304 (132, 768) |
| Remote Nunavut Community | 1486 (1018, 1690) | 1.82 (0.67, 3.29) | 1633 (1147, 1833) | 0.95 (0.33, 1.67) | 147 (122, 152) | 0.87 (0.33, 1.59) | 169 (96, 368) |
| **Full Cohort** | 2278 (1591, 2816) | 1.71 (0.64, 3.07) | 2447 (1741, 2959) | 0.85 (0.31, 1.49) | 169 (132, 188) | 0.86 (0.33, 1.64) | 197 (92, 491) |
| **Sensitivity Analysis: Hospital discharge for those started on empiric therapy who are Xpert®MTB/RIF negative**  **1 specimen analyzed with Xpert®MTB/RIF** | | | | | | | |
| Iqaluit Hospital Community | 5261 (3330, 6894) | 1.28 (0.46, 2.16) | 2960 (1501, 4625) | 0.71 (0.22, 1.24) | -2301 (-1585, -2631) | 0.57 (0.20, 1.12) | Saving |
| Remote Nunavut Community | 1486 (1018, 1699) | 1.82 (0.69, 3.15) | 982 (710, 1174) | 1.20 (0.45, 2.06) | -504 (-272, -581) | 0.62 (0.22, 1.24) | Saving |
| **Full Cohort** | 2278 (1580, 2662) | 1.71 (0.63, 2.85) | 1397 (913, 1837) | 1.10 (0.39, 1.97) | -881 (-581,-934) | 0.61 (0.21, 1.15) | Saving |
| **Sensitivity Analysis:Hospital discharge for those started on empiric therapy who are Xpert®MTB/RIF negative**  **3 specimens analyzed with Xpert®MTB/RIF** | | | | | | | |
| Iqaluit Hospital Community | 5261 (3302, 6959) | 1.28 (0.45, 2.19) | 3349 (1671, 5236) | 0.46 (0.14, 0.70) | -1912 (-1329, -2057) | 0.82 (0.31, 1.51) | Saving |
| Remote Nunavut Community | 1486 (1022, 1686) | 1.82 (0.71, 3.01) | 1061 (784, 1249) | 0.95 (0.35, 1.57) | -425 (-213, -488) | 0.87 (0.34, 1.51) | Saving |
| **Full Cohort** | 2278 (1585, 2719) | 1.71 (0.65, 2.96) | 1541 (1032, 2044) | 0.85 (0.30, 1.43) | -727 (-472, -790) | 0.86 (0.33, 1.54) | Saving |

* Negative incremental cost indicates "Savings" with Xpert®MTB/RIF strategy relative to the Status Quo strategy

**REFERENCES**

1. van't Hoog AH, Meme HK, Laserson KF, Agaya JA, Muchiri BG, Githui WA, et al. Screening strategies for tuberculosis prevalence surveys: the value of chest radiography and symptoms. PloS one. 2012;7(7):e38691.

2. Menzies D. Canadian Tuberculosis Standards. Public Health Agency of Canada, Ottawa, ON, Canada. 2014.

3. Alvarez GG, Van Dyk DD, Desjardins M, Yasseen III AS, Aaron SD, Cameron DW, et al. The feasibility, accuracy and impact of Xpert® MTB/RIF testing in a remote Aboriginal community in Canada. Chest. 2015;2015 Mar 19. doi: 10.1378/chest.14-2948.
